# A qualitative exploration of university student perspectives on mindfulness-based stress reduction exercises via smartphone app in Bangladesh

**DOI:** 10.1080/17482631.2022.2113015

**Published:** 2022-08-18

**Authors:** Munjireen S. Sifat, Naima Tasnim, Kirsten Stoebenau, Kerry M. Green

**Affiliations:** a The University of Oklahoma Health Science Center, Health Promotion Research Center, Oklahoma City, Oklahoma, USA; b BRAC James P Grant School of Public Health, Dhaka, Bangladesh; c Department of Behavioral and Community Health, University of Maryland School of Public Health, College Park, Maryland, USA

**Keywords:** Mindfulness, mental health promotion, digital health, global mental health, university students, Bangladesh, app

## Abstract

**Purpose:**

Mental health problems are proliferating, and access to mental health care is difficult due to barriers imposed by the COVID-19 pandemic in low-income countries such as Bangladesh. University students are susceptible to mental health concerns, given their unique stressors (i.e., academic pressure, new social environment). Mindfulness techniques can promote mental health , yet their acceptability has not been examined among Bangladeshi university students. These techniques can be used on a digital app, to decrease barriers to use.Qualitative methods were used to examine the acceptability of mindfulness among university students in Bangladesh. In-depth interviews (n = 12) were conducted to examine student reactions to linguistically (Bangla) and culturally adapted mindfulness exercises. Thematic analysis generated three themes (1) previous experience with mindfulness (2) positive responses to and (3) improvements to mindfulness exercises.

**Results:**

The results showed favourable attitudes towards the mindfulness content; students expressed positive psychological and physiological reactions. Students welcomed the concept of using these exercises on an app and felt it could overcomepast barriers to help-seeking.

**Conclusions:**

This evidence suggests the value of exploring the acceptability of an app with mindfulness exercises for mental health promotion through a larger-scale pilot study in university students in Bangladesh.

## Introduction

As the global mental health burden rises, particularly in the aftermath of COVID-19, mobile health (mHealth) can fill in the gaps where services may otherwise not be available. The global burden of disease attributable to mental disorders has risen in all countries in the context of major demographic, environmental, and sociopolitical transitions (Patel et al., 2018). The mental health of people has been severely impacted by COVID, particularly in low-income countries, where the additional stress exacerbates what mental health infrastructure exists (Kola, 2020). There is evidence of increased mental health distress symptoms and burgeoning uncertainty caused by the pandemic (Kola et al., 2021). In Bangladesh, one study surveying over 10,000 people found a high percentage of depression and suicide, 33% and 5%, respectively (Mamun et al., 2019). There has also been an increase in the suicide rate in neighbouring India, which is thought to be linked directly to COVID-19 (Kola, Kohrt, Hanlon, et al., 2021). The need for prevention efforts to curb clinical mental health problems cannot be understated.

These statistics are particularly troubling as people with mental health conditions have higher rates of disability and mortality when compared to people without mental health conditions (Pathare et al., 2018). For example, people with major depressive disorders, schizophrenia (and other psychotic disorders), and bipolar disorder have a life expectancy of 10–20 years shorter than the general population (Liu et al., 2017). The concern for mental health in low and middle-income countries is great, as mental health care services are scarce. Low-income countries often only have one psychiatrist to treat one to four million people (Chisholm et al., 2007; Lund et al., 2010; Patel et al., 2018). In Bangladesh, only 4% of all medical doctors have mental health training, and one psychiatrist is available for .07 of 200,000 of the Bangladeshi population, which ranks Bangladesh among the lowest rates of psychiatrists in the world (Giasuddin et al., 2015; World Health Organization [WHO], 2007). Further, general physicians rarely refer their patients to mental health specialists (WHO, 2007).

The Bangladeshi population may also face stigma surrounding accessing mental health services, as there is evidence that this exists in Asia. In Asia, there is documented widespread stigma surrounding mental health; however, few studies have examined the Bangladeshi views on mental health. One study in a University student sample in Bangladesh found that stigma was not significantly associated with reduced utilization of clinical mental health services; however, it is essential to note that the sample had low levels of stigma overall (Sifat, Tasnim, et al., 2022b). However, in a systematic review on this topic, it was found that a majority of the community does not recognize mental illness, that attitudes regarding treatment are negative, and that even those with mental illness do not see treatment as a priority (Hossain et al., 2014). Evidence from studies in the US suggests that Muslims may underutilize professional mental health services due to stigma (Ciftci et al., 2013; Din et al., 2017). There are also documented cases of Muslims from LIC, such as Pakistan, preferring to go to religious healers to treat mental health conditions, believing their symptoms stem from problematic faith (Farooqi, 2006; Irfan et al., 2017). As Bangladesh is a Muslim-majority country, increasing methods less prone to stigma is essential, and interventions may be well suited for this population.

The population impacted by suicide the most in Bangladesh is young adults (Arafat, 2019). Globally, mental health problems, such as depression, arise during young adulthood (McGorry, 2011). People in college are particularly vulnerable, as they fall into this young adult age group and are also in a period of adjustment, facing stressors related to university life, such as academic pressures, living away from their families, and being in a new environment (Eisenberg et al., 2011). As reported by recent studies, Bangladesh university students suffer from high rates of depression, ranging from 47.5% (Sayeed et al., 2020) to 69.5% (Islam et al., 2020).

Behavioural strategies and mindfulness techniques are often a part of preventive interventions that promote mental health (Regehr, Glancy, and Pitts, 2013). The practice of mindfulness is widely used for mental health promotion and training in regulating attention (Barnes et al., 2017). The popularity of mindfulness training can be partly explained by its perceived status as a skill rather than a mental health intervention (Galante et al., 2018). A meta-analysis examined the effect of mindfulness interventions in college students and found that mindfulness led to reduced depression, anxiety, stress, general psychological distress, better social-emotional skills, self-perceptions, and academic adjustment. (Conley et al., 2013). In general populations, recent systematic reviews of meta-analyses have found that mindfulness interventions consistently led to better mental health outcomes (i.e., lower stress, higher mindfulness, a better quality of life; Goldberg et al., 2022; Taylor et al., 2021). One review focused on mindfulness-based self-help, as is explored in the current study, and found that these led to significantly lower depression levels, anxiety and stress symptoms, higher mindfulness levels, and quality of life/well-being levels than control conditions; however, the effect size was small (Taylor et al., 2021). These findings demonstrate the importance of supporting mental wellness and that there are well-researched methods that are effective at doing so. Mindfulness has also been used effectively in a mHealth platform among university students in Asian countries, such as China, to reduce anxiety symptoms during COVID-19 (Sun et al., 2022). These findings are promising, as mHealth for mental health is one method that can be used to allow access to care in low-income country settings like Bangladesh.

One method that shows promise for promoting mental health is using mHealth. The concept of mobile health (mHealth) refers to mobile computing and communication technologies in health care and public health (Free et al., 2010). In low and middle-income countries (LMIC), the use of mobile phones is widespread (Kola, 2020), as is the use of smartphones (phones using mobile internet networks; Kale et al., 2016; Kola, 2020). Evidence supports that mHealth can increase the likelihood of health interventions being delivered to otherwise hard-to-reach LMIC populations (Marcolino et al., 2018). Other advantages of mHealth are convenience, ease, cost-effectiveness, scalability, personalization, and “the ability to send time-sensitive messages with an ‘always on’ device” (Whittaker et al., 2016). In a sample of university students in Bangladesh, one study found that 26% (n = 82/311) of the sample currently use digital health for mental health promotion (Sifat, Saperstein, et al., 2022). The same study found that the majority of the sample (76.3%) reported that they were either somewhat or extremely likely to use an app for self-paced mindfulness or non-clinical practices to better their mental health. Further, mHealth can reach populations who would otherwise not engage with traditional health services (Hamine et al., 2015). One study found that higher non-stigma-related barriers to utilizing mental health care (such as not having transportation to access a mental health clinic) were significantly associated with using digital health for mental health Sifat, Saperstein, etal., (2022). It can also tackle social stigma, as accessing an app on a mobile phone can be done privately without fear of others watching. These benefits can contribute to early intervention, which is essential, as the earlier a person can manage stress and depressive symptoms, the better their overall health outcomes will be (Bukh et al., 2013).

Apps have been shown to successfully increase mental health wellness outcomes and decrease symptomatology of mental health problems. Mobile apps are beneficial as they can be personalized (Gustafson et al., 2014), visually engage the user, track progress, and be self-paced (Bricker et al., 2014; Luxton et al., 2011). A systematic review of over 5,646 abstracts published between 2008 and 2013 found eight papers describing five apps targeting depression, anxiety, and substance abuse that met their inclusion criteria (Donker et al., 2013). Results showed, across the studies, significant reductions in depression, stress, and substance use. A recent meta-analysis of meta-analyses suggests that psychological intervention content delivered via a web- or mobile app can be as efficacious as a face-to-face treatment for depression (Andersson et al., 2014; Andrews et al., 2010; Cuijpers et al., 2010). Mental health apps are popular among young adults as they provide autonomy to the user, which young adults rate as highly important in their consideration of motivations for behaviours (Fuller-Tyszkiewicz et al., 2018; Wilson et al., 2011) and offer great promise to under-resourced settings, like Bangladesh.

Bakker et al. (2016) developed guidelines to ensure the proper development of mental health apps based on a systematic review of existing apps and using Fogg’s behaviour model. Among mindfulness apps, the BlueWatch app stands out as it was created by a team of researchers, psychiatrists, and psychologists, all of whom have expertise in mHealth delivery of interventions for depression, and fulfils 14 of Bakker’s criteria. The app’s creation utilized user-centred design, was grounded in theory and has been tested for end-user and expert usability (Fuller-Tyszkiewicz et al., 2018). One app component includes short audio activities that engage users in mindfulness activities. The intended outcomes of the app are to improve the well-being and resilience of adults experiencing depressive symptomatology.

As there is evidence that interventions that work for some populations may not be effective for others, it is essential to use a culturally adaptive framework to assess BlueWatch in a Bangladeshi population. Barrera and Castro (2006) present a heuristic framework for the cultural adaptation of interventions. There are three stages: 1) information gathering, 2) preliminary adaptation tests, and 3) adaptation refinement. Information gathering encompasses a literature review and conducting quantitative surveys to understand the demographic characteristics and preferences of the target population. It also involves qualitative research with potential participants within the target population or interviewing experts in the field who have experience working with the target population (Barrera & Castro, 2006; Cabassa & Baumann, 2013; Dumka et al., 1998). This study aims to address the first component of this framework, information gathering, to examine if a linguistically and culturally adapted version of the mindfulness exercises used in BlueWatch is acceptable in a Bangladeshi University population. Specifically, this study seeks to understand the feasibility and acceptability of mindfulness exercises on a mHealth app platform among current Bangladeshi university students through semi-structured interviews regarding specific mindful exercises with 12 students. The research questions being explored in this paper are: “*How do students a) respond to mindfulness exercises for university students in Bangladesh?”* and “*How do Bangladeshi university students describe their perceptions of engaging in mindfulness exercises on a smartphone app?”*

## Methods

### Sampling and recruitment

Participants (n = 12) were purposively recruited from a list of university students who participated in an online survey about university student attitudes towards mental health in Bangladesh. In that survey, students noted if they consented to be contacted for a potential in-depth interview to discuss the topic of mental health. Participants were stratified by gender and randomly selected from within gender groups. The eligibility criteria for participants included 1) being 18 years or older, 2) currently enrolled in a university within Bangladesh, and 3) and had access to the internet to conduct a Zoom interview.

### Conceptual framework

**Figure 1.**
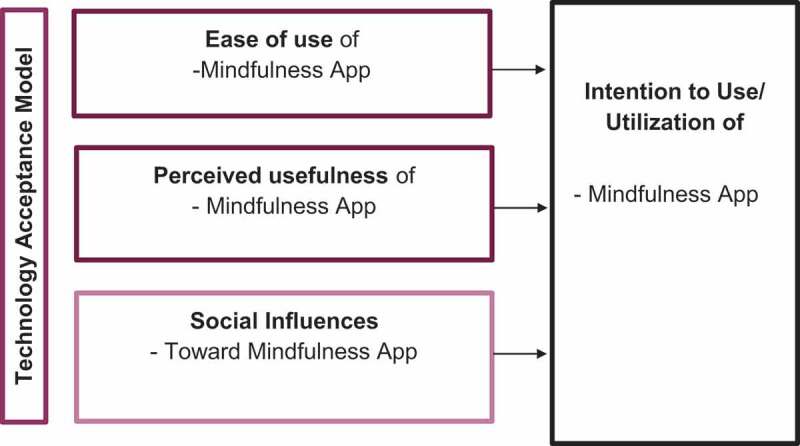
Conceptual model using the technology acceptance model.

This study was guided by the Technology Acceptance Model (TAM), which helps researchers understand how users adopt and use emerging technologies (Portz et al., 2019). According to the conceptual model (see, Figure 1), a user’s perception of the usefulness (i.e., the perceived benefits) and the ease of use of technology determines their willingness to use it. TAM posits that people’s differences and social influences shape their perceptions of usefulness and ease of use. Accordingly, the interview guide included questions that probed the concepts of perceived usefulness, ease of use, and social influence towards using mindful exercises.

### Procedure

Semi-structured interviews (n = 12) were conducted by two people, the lead author, and a research assistant. The lead author was fluent in Bangla, and the research assistant was a recent graduate of a university in Bangladesh and also a native Bangla speaker. The research assistant led the interviews in Bangla, using an interview guide developed for this study. Interviews lasted between 40 and 65 minutes and were an average of 45 minutes. Ethical approval for this study was given by the University of Maryland’s Institutional Review Board (IRB number: 1,656,046–3) and administrative faculty within the Bangladesh universities.

#### Cognitive interviews

Prior to the in-depth interviews, cognitive interviews were conducted to ascertain the cultural appropriateness of the language that was to be used during the in-depth interviews. During these cognitive interviews, participants were also asked how they defined mental health and to explain their perception of what specific questions were asked. Items that cognitive interview participants had trouble understanding or that were culturally inappropriate were adapted so they could be easily understood. For example, one item asked, “How often did you feel down, depressed, or hopeless in the past two weeks?” Upon hearing this item, all participants asked interviewers to clarify what “feeling down” meant and said that this phrase was not used in Bangladesh to denote feelings of negative affect. They confirmed that the item was clear to them if the phrase “feeling down” was removed. After completion of the cognitive interviews, the interview guide was adapted into a finalized form.

#### Semi-structured interviews

During the interview, participants were given an overview of mindfulness and were asked to follow three mindful exercises. After each exercise, participants were asked how they felt after completing the exercise and during the exercise, what they liked and would like to change about the exercise, and if they could see themselves using an app with exercises like these in the future. After the final exercise, participants were asked if they would recommend an app with these exercises to a friend and the reasons behind why they would or would not use an app similar to this.

#### Exercise 1

The first exercise was 2.5 minutes long and was designed for participants to train their observant side and to focus on being present with their surroundings. For example, participants were given the following directions “*I want you to focus on your preferred hand. Spread this hand so the fingers aren’t touching and the hand is flat. Lay this flat hand on a surface such as a table. Focus on the appearance of your hand.”* The exercise included questions that prompted the participant to closely consider themselves in the moment, such as, “*Focus on how your hand feels right now. Does the surface feel cold to touch?”*

#### Exercise 2

The second exercise used breathing techniques to diffuse negative thoughts and lasted four minutes. It started with an introduction, “*Using the breath to focus is another effective way to get back to the here and now. It also helps you to slow down, relax and diffuse negative thoughts by controlling where you direct your attention,”* and then guided participants through the exercise. “*When you are ready, take some nice big, deep breaths in through the nose and out through the mouth. And [breathe] in. Notice how your body softens as you do this. On the next out-breath, slowly close your eyes.”* The exercise provided imagery for the participant, “*think of your breathing as waves going into shore as you breathe in*.”

#### Exercise 3

The last, and longest exercise (9 minutes), guided participants through a body scan to de-stress. It too began with a brief introduction of the exercise, “*The present exercise will walk you through a relaxation technique that seeks to remove tension from your whole body. We will systematically work our way through focusing on different parts of your body in order to bring relief*.” The exercise then went through a full-body scan, starting at the toes, “n*ow directing your attention to the toes of your feet. Tune in to the sensations in your toes.”*

### Qualitative analysis

Interviews were audio-recorded, transcribed verbatim, and translated from Bangla to English before being uploaded into Dedoose for thematic analysis. The interviews were translated and transcribed into English by the research assistant and checked by the lead researcher for fidelity purposes. Thematic analysis allowed for flexible and detailed data synthesis (Braun & Clarke, 2006; Nowell et al., 2017). Thematic analysis allows researchers freedom not to be wedded to theoretical commitments of grounded theory (Braun & Clarke, 2006). For this paper, thematic analysis was used with a contextualist method, which encompassed characteristics of both essentialism and constructionism. The purpose of thematic analysis in this study was to “provide a rich thematic description of the entire data set,” contributing to readers having “a sense of predominant or important themes” (Braun & Clarke, 2006). Braun and Clarke (2006) outline a six-step method for conducting thematic analysis, which was utilized for this analysis, including: becoming familiar with the data, developing and applying deductive and inductive codes, searching for themes in the relationships between codes, reviewing and refining themes and underlying code relationships, naming themes, and writing results. After each individual interview, two research team members debriefed and engaged in reflexive journaling (Nowell et al., 2017). These notes taken during reflexive journaling were reviewed before themes were developed with the final data. The themes were created by one research member and reviewed by another to ensure fidelity (Nowell et al., 2017).

## Results

Table I shows the demographics of the sample. Participants were, on average, 22.7 years old. There were participants from first, second-, and third-year students and students who were in their fourth year of school or beyond. Most students in this sample pursued a Bachelor’s degree, though Master’s students are also represented. The majority (70.0%) of students interviewed felt they could use support for their emotional or mental health in the past year and were moderately stressed. Three students reported past suicidal ideation. Almost all students owned smartphones, and 70.0% used mHealth for physical health. Almost half (40.0%) of the sample reported using digital health for their mental health, for example, following online meditation videos.

**Table I.** Participant demographics n = 10 (demographics missing for n = 2).

	%/ M (SD)
Age (in years)	22.7 (1.83)
Gender	
Male	40.0%
Female	60.0%
Year/Semester in School	
1st-3rd/First year	30.0%
4th-6th/Second year	20.0%
7th-9th/Third year	10.0%
10th-12th/Fourth year	0.0%
12^th^ +/ Fourth year +	40.0%
Degree of Study	
Bachelors (BS, BA)	70.0%
Masters (MPH, MBA)	30.0%
Perceived need for mental health support (past year)	70.0%
Perceived Stress (0–16)	10.2 (3.99)
Suicidal Ideation (ever)	30.0%
Smartphone ownership	90.0%
Use of mHealth (not including for mental health)	70.0%
Use of mHealth for mental health	40.0%

Note: Though the total sample size is n = 12, only 10 participants provided demographic information

First, it should be noted that, though mindfulness was defined technically and participants were given examples of different forms of mindfulness, it was evident from student responses that the sample thought of mindfulness primarily as a deep breathing and meditation exercise.

**Figure 2.**
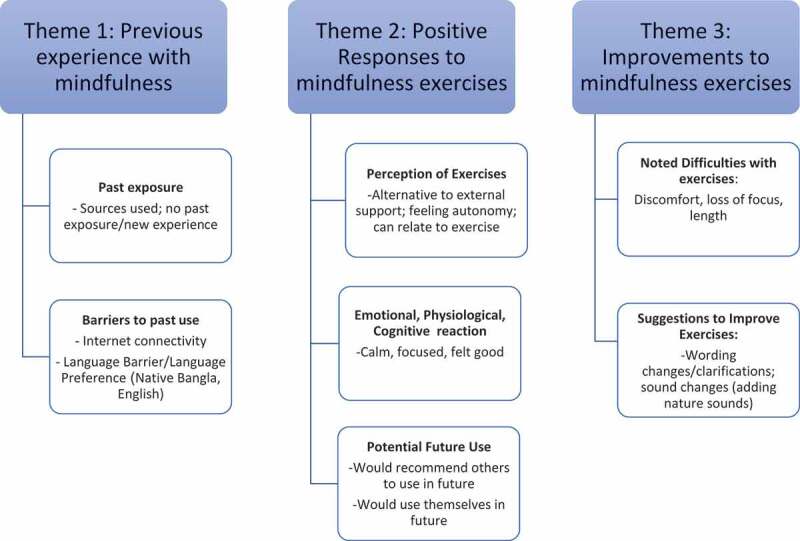
Thematic map.

Three major themes and seven subthemes emerged from the interview data. The first theme encompasses participants’ experience with mindfulness (if any); participants noted if they had ever participated in mindfulness exercises before, what sources they used, and why they did or did not use these practices consistently. The second theme was titled positive responses to mindfulness exercises; subthemes included perceptions of how mindfulness exercises were beneficial to them, describing the emotional or physiological reaction they had to the exercises (i.e., feeling calm or focused), and their potential for future use of these exercises for themselves, or a friend. The third theme relates to proposed improvements for the exercises; participants suggested changes they thought would improve the exercises or expressed difficulties with the exercises that may warrant adaptations. See, Figure 2 for the thematic map created.

### Theme 1: previous experience with mindfulness

Most of the students interviewed had at least some experience with mindfulness activities. Participants noted barriers they encountered to engaging in mindfulness activities in the past. Participants identified the role of language as a barrier to participation in mindfulness, given that the language in an exercise did not align with their preference. The majority of the sample preferred their native language of Bangla but also noted the importance of having a choice to pick between Bangla and English as the best option for the college student population at large. Participants noted whether the experience felt beneficial enough to want to continue doing similar exercises in an app format and, further, the process by which they would promote the use of such an app to their friends. The interviews also shed light on difficulties that participants faced while guided through the mindfulness exercises. These findings highlight the overall feasibility of using an app for mindfulness.

#### Past exposure to mindfulness

Those who had previous experiences with mindfulness (n = 9) explained the methods and extent to which they interacted with mindfulness; some participants shared why they engage in these activities, for example, wanting to de-stress. Of the participants who use mindfulness techniques, most (n = 4) cited doing breathing exercises (the main focus of exercise 2, though it is integrated into exercise 3 as well) as a reaction to a feeling stressed. Participants became aware of exercises in varying ways, some sought out ways to cope with stress for themselves, heard about the exercises from other people, and one mentioned seeking information to help a friend suffering from depression. The following are illustrative quotes from the interviews.

*“Actually, I tried this kind of exercise on YouTube focused on deep breathing and releasing. Let me tell you a funny story, one day I fell asleep while doing these exercises. Breathing exercises are great for both health and mind. I still try to do it, not every day, but whenever I feel a little bit stressed.”*

*“I have seen and done simple breathing exercise several times. However, an instruction helps a lot, I guess. Actually, my friend was a clinical mental patient who was suffering from depression. When he talked about it, I didn’t know how I should react or what I should suggest him to do. Then I googled it and found out about this exercise.”*

#### Barriers to past use

Participants who had previous experience with mindfulness noted various barriers to sustained use. They expressed wanting to use mindful activities to relieve stress but that they were unable to find quality materials with guided meditations. Participants (n = 4) reported using YouTube as a primary source for mindfulness exercises. However, they had issues finding quality exercises for mindfulness on this platform, as illustrated by one participant who explained, “I tried a couple of meditation videos on YouTube which were boring so I quit watching them.” Another participant noted that it was sometimes hard to access YouTube; they said, “I have done some meditation sessions by myself on YouTube. However, I could not do it regularly as YouTube wasn’t available all the time.” Bangladesh is prone to electrical shortages, internet systems are sometimes not readily available, and YouTube videos can be restricted if deemed offensive by the government.

Another significant barrier to past use, and a key factor to consider with respect to feasibility, is the language of the mindfulness exercises. Participants noted that audio for meditation is hard to find in the native language preference of Bangla. One participant demonstrated this, saying:

… when we try to do this exercise in YouTube, maximum instructions are given there in English. If someone who’s not very good at English uses it, [they] can find it hard to understand the meaning of some words. This can cause tension rather than finding peace. There’s no point of thinking of a dictionary if you’re using a relaxation method.

Most participants said they would prefer to have the option of choosing the language of the app. Participants noted that some people might be more comfortable listening to the exercises in Bangla; one participant said, *“people can choose the language on their preference. I personally like Bangla as it’s our native language and it’s easier for us to focus in this language.”* Though the majority of the sample said they would prefer an app in Bangla for their own use, they noted that other people might want to use English and highlighted the importance of being able to choose the language of your choice. One participant summarized this by saying:

It will be better if there are two versions. If we think in the context of Bangladesh, there are two types of people, some of them will prefer Bangla and some of them will English. As the mental health is really an important topic for every Bangladeshi people, so it’s better to have both options.

Barriers to using mindfulness techniques seem to stem from the lack of access and availability of quality exercises in participants’ language of preference. Participants (n = 9) noted that their language of choice when utilizing guided mindfulness exercises is their native language of Bangla. However, some participants (n = 5) also noted that this might not be the case for all university students in Bangladesh; when thinking about the broader population who may use an app with these exercises, participants advocated for having the option to choose between Bangla or English. Though there was mention of using YouTube to follow meditation videos, participants had difficulty finding videos in Bangla and had limited access to YouTube. If an app was developed or an existing app adapted for a Bangladeshi population, students would be able to download mindfulness exercises to use at any time and access Bangla mindfulness exercises whenever they wanted.

### Theme 2: positive responses to mindful exercises

Participants gave a detailed description of their perception of how they felt during the exercises. For example, many participants enjoyed the feeling that they could connect to what the exercise guided them to do. Other participants did not explain the mechanics behind why they had a positive experience but did share the positive emotions they were feeling throughout and after participating in the exercises. These subthemes were interrelated, as participants would explain that they felt calm during the exercise (a physiological reaction) and that feeling calm was the reason they had an overall positive experience. The overwhelming majority of responses to the mindfulness exercises were positive. However, some participants faced difficulties and noted suggestions for improving the exercises (explored in theme 3).

#### Perceptions of mindful exercises

Participants explained their perceptions of the mindfulness exercises. Some described that an app could help with mental health maintenance, given that many are not able to see a professional whenever they may want, saying “As we can’t go to a professional every other day, I think this app will be very effective if we get to do the exercises with these types of instructions.” Another participant discussed the benefits of having an app with mindfulness exercises given the inability to seek informal help, “As we can’t open up with someone all the time or can’t find the solution for our mental health problem, then this app can be effective to be in practice.” Others noted that they liked how the exercises caused them to focus in general and specifically focus on themselves in ways they had never done before. One participant described, “I like the way it focused on different body parts step by step as we usually don’t notice every little detail about ourselves.” They enjoyed connecting to what the exercises guided them to do; for example, participants could easily connect their breath to waves, as they could visualize the waves effortlessly. Illustrative quotes of the participants are below.

What I found new here is to connect my imagination with waves. I have actually watched a video of ocean last night or the night before that. The video was taken by a drone. So, I was actually imagining the blue ocean and the shore while breathing. I could connect it with my breathing process, and at one point I could feel that I was [in] the ocean.

Participants reported many mechanisms in with they felt the exercises impacted them. They noted being able to visualize and focus on the task the exercise guided them to do. When they visualized the exercises, participants felt they gained more focus while doing the exercises and afterwards since they felt more relaxed. One participant said,

Well, it’s hard to tell how beneficial was it by doing the exercises just one day. However, what I noticed from today’s experience are it helps to feel light and to keep focus on a specific thing. For example, when I started this interview, I wasn’t fully focused, but once I started doing the exercises, I realized that my capability of focusing on a specific thing was getting better. I feel like I can concentrate better now.

#### Emotional, physiological, and cognitive reactions

After each exercise, in response to asking how participants were feeling, many participants described what emotions they felt. Some participants mentioned feeling at peace and relaxed; some mentioned feeling relieved of stress. Some participants described their physiological response to the exercises, saying their bodies felt relaxed, and their heartbeat was slowed due to the exercise. However, not everyone felt a change, and one participant noted that they did not feel any impact, stating, “I feel okay; I don’t feel any different.” Examples of what the participants said follow:

It was beneficial for me. I was feeling a bit relaxed and lightheaded. When we feel relaxed, our heartbeats normally. At first, my heartbeat was really fast, but I think it’s stable now. If these exercises can make my heart stable, then they can make others as well. And this is a good thing.

Well, as I mentioned that I didn’t know anything about this interview before. I was feeling stressed whether I would be able to say anything or not, but after doing these exercises, I am feeling like all of my tensions just went away.

Overall, while explaining how they felt during or after the exercise, participants reported positive physiological reactions, less tense, calm, and relaxed. However, it is essential to note that some participants noted discomfort during the exercises, too; these comments were covered in depth in subtheme 3.1.

#### Potential future use

Participants noted that they would use and recommend an app with mindfulness exercises in the future and to their friends. Some participants indicated wanting to use such exercises reactively to mental health problems in order to aid them in getting relief from anxiety, depression, and stress. Others wanted to use mindfulness as a mental wellness maintenance tool, for example, to help them with relaxation and sleep or to keep negative thoughts away. Two participants said that doing the exercise would be a sort of distraction from other anxiety-related thoughts:

I think these exercises will work well to get relief from anxiety. For me, I think it has worked really well to keep my mind busy on focusing a specific thing by keeping all the negative thoughts away.

If I am mentally stressed or depressed, I think this type of exercise can help me to get some relief. Sometimes I lost my focus while doing something. So, if I can learn how to keep my focus on a specific thing through an app, it will be very beneficial for me. On top of that, if I can forget about my mental stress or ignore all the negative thoughts for some time, it will be really helpful.

Another participant noted that these exercises could work as an alternative to using entertainment as a distraction:

In our daily lives, we have a lot of work and study pressure. At this time, if we don’t have enough time to see a movie or series, then these type of short meditation sessions can help us to feel more relaxed. Moreover, if you could add some music in the background, it would be easier for us to keep our focus.

One participant even mentioned that doing these exercises could promote sleep:

Honestly, I almost fell asleep. I am feeling relaxed. Actually, I was tensed last night so couldn’t sleep properly, and now I’m thinking that if I did this exercise yesterday, I could have slept well.

Though all participants (n = 12) indicated wanting to use an app with mindful activities in the future, they had more specific reasoning regarding endorsing it to others. Many participants said that they wanted to try the app first, and if they felt it was beneficial to them in overcoming their problems, only then would they recommend it for their friends to use. Participants imagined how they would tell their friends about the app; most said they would gradually introduce the app; for example, they would first share their mental health struggles and then tell their friends how the app helped them. Others said they would tell specific friends who have talked to them previously about wanting help to promote their mental health. One participant felt that, by having a concrete app to share, it would be easier for friends to use that for mindfulness exercises instead of searching for themselves. Quotes from participants are shown below.

At first, I’ll try it by myself and see if it works for me or not. I can discuss it with my friends when we hangout and maybe one day I’ll try to make them practically focus on this matter. If they feel interested, then they’ll definitely try it out. So, I’ll try to convince my friends to use this app in the middle of our conversations when we go out. First I’ll use it practically, then suggest my friends to use it.

Well, if I talk about my gym buddies, there were times when we did exercises related to mental health which didn’t have any guidelines. So, if I want to start, I’ll start with those who believe that they need to work on mental health and those who doesn’t think like that, I can start talking to them about this topic gradually.

Participants noted they would only endorse the product if it proved effective for them. They also offered nuanced approaches depending on their relationship with their friend. If they knew that their friend would be open to a sustainable solution, they would be able to bring up the topic of this app easily, if they were in the midst of a mental health crisis, they would not bring up the app right away until they felt their friend was open to it.

### Theme 3: improvements for the mindfulness exercises

The third theme regards improvements to the mindfulness exercises that the participants identified. Recommendations included suggestions for a logo, changes to content, for example, clarifying specific wording in the guided exercise, or changing parts of the audio to include soothing music or sounds. Students identified the language of their preference, the majority preferring their native language of Bangla, but also saying having an option to pick between Bangla and English would be best for the college student population at large, as was discussed in sub-theme 1.2. Other improvements stem from the difficulties participants faced while guided through the mindfulness exercises.

#### Noted difficulties with the exercises

We found that some participants had difficulty listening to the exercises. Some expressed that the level of detail and length of the exercises were not appropriate for someone who was a beginner to mindfulness activities; they felt this led to a lack of focus during the exercise. This was particularly true during exercise 3, the body scan, given the length of the exercise. Participants also felt irritated with themselves for their lack of focus. Also, specific to exercise 3, one participant expressed discomfort when the guided body scan asked them to focus on their feet. Another expressed feeling odd when focusing on their forehead and neck during exercise 3. Importantly, though participants experienced discomfort, they nonetheless expressed positive feelings towards the exercises.

Well, I feel heavy, you know. I think it’s too much detailed for a starter. The interesting thing is when it mentioned to focus on my neck and shoulder, I felt like some was strangling me and trying to suffocate me. I don’t know why I felt that. And when it said to focus on my forehead, I felt like I’ve opened a third eye. But I’m feeling a bit heavier right now, and if I go to bed now, I think I’ll fall asleep.

When I brought focus on my upper body parts, I started to feel heavy then. I was struggling while focusing on my lower body parts. I started to feel a bit irritating as I couldn’t focus.

Interestingly, when participants were asked whether they would like to change the exercise in any way, the overwhelming response was to say that the exercises did not require any changes. However, as shown in the illustrative quotes above, some recommendations were explicitly stated, and others were illuminated in their responses to the question, “how did you feel during the exercise ?” The explicit changes participants recommended are described in detail in theme 3. Overall, the main difficulty that participants seemed to face was drifting focus, though it is noted in the instructions throughout the exercises that it is normal if one’s mind wanders throughout the exercise. Part of the difficulty of loss of focus had to do with the length of the exercises. This highlights the need to allow participants the option to choose their preference of the duration (short, long) and level (beginner, intermediate, advanced) at which they would like to engage with the app.

#### Suggestions to improve mindfulness exercises

Participants noted multiple methods to improve the mindfulness exercises. Recommendations included suggestions for a logo, changes to content, clarifying specific wording in the guided exercise, or changing parts of the audio to include soothing music or sounds. Participants discussed content changes that they felt would be beneficial, such as clarifying the guided instructions’ wording or changing the length of exercises, such as shortening the body scan. They also noted design preferences, particularly regarding aesthetic preferences- using attractive colours and fonts, user-friendly, and minimized advertisements- for a potential app interface. Further, multiple (n = 3) interviewees recommended adding nature sounds (sounds of waves). Participants drew attention to where they felt there were gaps in the exercises; for example, in the third exercise, one participant felt more focus should have been put on the upper body during the body scan.

As it creates an impact on the whole body, I think every point of this exercise is important. However, I feel like it needs to add more focus on the neck and shoulder areas.

I think if you could add ocean wave sound in the background, it would have been really great. That’s my personal opinion.

[The app should be] a user-friendly app, [with] visible letters that attracts people, use attractive colors or font, [and] reduce advertisements.

Given that multiple participants explicitly mentioned that adding audio of ocean waves throughout the second exercise would be beneficial, this should be included in future pilot testing for the app development. Participants brought up aspects of visual app design, though they were not asked explicitly; this highlights the need for users to find app interfaces aesthetically pleasing and easy to use, highlighting the necessity for an app developed with user-centred design.

## Discussion

Overall, there is much promise for using mindfulness activities in this sample of Bangladeshi university participants. Results suggest that university students would be interested in using mindfulness activities to promote their mental health. In addition, participants suggested they would use an app with such exercises in the future and even recommend it to friends if they thought it was helpful. The findings highlight that an app with quality, empirically driven mindfulness content in participants’ native Bangla language could build a bridge from the past barriers faced by participants.

Additionally, using an app for mindfulness exercises would allow participants to download content to their phones rather than rely on the internet, which participants report having issues within Bangladesh. Participants also provided valuable suggestions for improving the mindfulness exercises, including noting areas they felt were difficult for them to follow, the importance of the duration of exercises, and noting the importance of appropriate language for the exercises. The drive to have an app in one’s native language has been seen in past research (Kiropoulos et al., 2011), as is the option of having language preferences that the users can control (Torous et al., 2019), though the acceptability of a Bangla app for mental health has not been examined before. Participant responses highlighted the desire to have a customizable app.

Second, this study explored the acceptability of a mindfulness app by identifying the perceived benefits of its use. Delving into each exercise, participants noted feeling a positive effect from the exercises. Positive experiences varied significantly; some participants noted that the possibility of having access to mindfulness exercises on an app would be beneficial given they are not able to see a professional or informal help readily, and believe the app would reduce this barrier of access by bridging this gap in terms of mental health promotion. Others noted that the mindfulness exercises helped them to feel relaxed, calm, and focused. Some mentioned they would use this as a preventative measure to promote their mental wellness, and the majority claimed they would use it in response to stress. These findings support past research that finds mHealth effective for mental health promotion (Donker et al., 2013) and used for stress management efforts (Bukh et al., 2013).

Of the three exercises, exercise 2, “breathing to diffuse negative thoughts,” was overwhelmingly viewed most favourably- no participants had difficulties following this exercise. However, a couple did suggest including sounds of ocean waves in the audio. This exercise also had the most mentions of feeling relaxed and refreshed afterwards. The third exercise, “de-stressing with a body scan,” was viewed least favourably of the three, although overall, it was viewed positively. Participants noted that the length of this exercise (approximately 9 minutes) was longer than desired and that it was hard to concentrate on the task. One response, unique to the participant, noted feeling uncomfortable focusing on their feet, given the lack of cleanliness of the surrounding area in their room.

### Limitations

Several limitations should be considered regarding these findings. Participants were recruited after taking a survey regarding mental health attitudes. As such, this sample may already view mental health positively and, thus, would welcome the possibility of using an app to help with their mental health. Indeed 70% of the sample reported needing support for their mental health, and 40% already reported having used mHealth for their mental health in their lifetime. This may not be true of the general student population, as there is evidence that mental health stigma in low-income countries in Asia is high (Giasuddin et al., 2015). This study sought first to examine the messaging of mindfulness exercises and second, examine attitudes towards using a guided mindfulness exercise with similar messaging on an app-based platform. However, when conducting interviews, only the message was able to be tested. As neither wire diagrams nor a pilot app was provided to participants, their responses regarding whether or not they would use an app with similar activities were based purely on their familiarity with apps and their imaginations of how a similar app might work. Examining the acceptability and generating feedback about mindfulness messaging is just the first step of the cultural adaptation process outlined by Barrera and Castro (2006) of information gathering. Next, steps must include the preliminary adaption tests based on these findings and adaptation refinement. This study provides preliminary evidence that mindfulness exercises and their availability on an app are viewed favourably by a university student population in Bangladesh. It is a stepping stone for a larger-scale study to assess the acceptability and feasibility of such an app truly.

### Implications for future research and practice

Given the positive feedback provided by the sample after these exercises, future research should develop and pilot-test an app with mindfulness messaging for the university student population in Bangladesh. Given the ample amount of apps already in existence, it is feasible to culturally and linguistically adapt evidence-based apps for this purpose. This formative research utilized a study sample of n = 12 participants, which allows the researcher to establish a close relationship with the respondents, enhancing the validity of in-depth investigations (Crouch & McKenzie, 2006; Vasileiou et al., 2018). As a next step, it would be beneficial to assess the acceptability of mindfulness in a larger sample size and utilize sampling methods to test the acceptability among different subgroups that may have differing opinions towards mindfulness or mental health (i.e., gender). As the cultural and linguistic adaptation of BlueWatch was viewed positively in this sample, except for participants expressing discontent with the body scan exercise, given the length and other feelings of discomfort, it may be appropriate to use BlueWatch as a template for the design of such an app. Since participants report motivations for use as both preventative and early intervention to manage stressors, they could choose how often they want to be prompted to use the app. As participants noted the length of exercises as a factor in considering whether or not they would engage in a mindfulness exercise, students could first be prompted to measure their stress level and second asked how much time they have available to complete a mindful exercise. Based on these answers, the students would then be directed to a short, medium, or long exercise.

## Conclusion

This research supports the narrative that mindfulness exercises can be appealing on an app and that it is appealing to a university student population in Bangladesh. Based on these in-depth interviews, we can conclude that students perceived the benefits of mindfulness exercises and wanted to use quality exercises in their native language of Bangla. Given the high prevalence of mental health problems in Bangladesh (Mamun et al., 2019) and that young adult college populations are disproportionately impacted (Islam et al., 2020; Sayeed et al., 2020), these results that a mindfulness app is acceptable for such a population is quite promising.

Similar to past research, this sample noted the appeal of having a personalized app (Gustafson et al., 2014) that is aesthetically pleasing (Bricker et al., 2014). Students report they would not only use an app with mindfulness exercises if it existed but that they would also refer their friends to use such an app if they felt it benefitted them. To our knowledge, this is the first study to examine the acceptability of mindfulness practices in a Bangladeshi university student population. Further research should consist of app development for pilot testing of messages and user-centred design. Should this further testing show promise, the app should be refined and shared across universities in Bangladesh.
